# Investing in updating: how do conclusions change when Cochrane systematic reviews are updated?

**DOI:** 10.1186/1471-2288-5-33

**Published:** 2005-10-14

**Authors:** Simon D French, Steve McDonald, Joanne E McKenzie, Sally E Green

**Affiliations:** 1 Australasian Cochrane Centre, Institute of Health Services Research, Monash University, Monash Medical Centre, Locked Bag 29, Clayton, Victoria, 3168, Australia

## Abstract

**Background:**

Cochrane systematic reviews aim to provide readers with the most up-to-date evidence on the effects of healthcare interventions. The policy of updating Cochrane reviews every two years consumes valuable time and resources and may not be appropriate for all reviews. The objective of this study was to examine the effect of updating Cochrane systematic reviews over a four year period.

**Methods:**

This descriptive study examined all completed systematic reviews in the Cochrane Database of Systematic Reviews (CDSR) Issue 2, 1998. The latest version of each of these reviews was then identified in CDSR Issue 2, 2002 and changes in the review were described. For reviews that were updated within this time period and had additional studies, we determined whether their conclusion had changed and if there were factors that were predictive of this change.

**Results:**

A total of 377 complete reviews were published in CDSR Issue 2, 1998. In Issue 2, 2002, 14 of these reviews were withdrawn and one was split, leaving 362 reviews to examine for the purpose of this study. Of these reviews, 254 (70%) were updated. Of these updated reviews, 23 (9%) had a change in conclusion. Both an increase in precision and a change in statistical significance of the primary outcome were predictive of a change in conclusion of the review.

**Conclusion:**

The concerns around a lack of updating for some reviews may not be justified considering the small proportion of updated reviews that resulted in a changed conclusion. A priority-setting approach to the updating of Cochrane systematic reviews may be more appropriate than a time-based approach. Updating all reviews as frequently as every two years may not be necessary, however some reviews may need to be updated more often than every two years.

## Background

When people make decisions about health care they should have access to the most up-to-date and reliable evidence. Cochrane systematic reviews aim to provide healthcare professionals, consumers and policy makers with the 'best available' and most up-to-date evidence on the effects of healthcare interventions. One of the advantages of an electronic publication such as *The Cochrane Library *is that reviews are replaced with an updated version as this new evidence becomes available or mistakes are identified [1]. This differs from print journals where readers do not necessarily know if they are accessing the most up-to-date systematic review available. A reader of a Cochrane review will therefore expect that the information is up-to-date.

Since evidence used to inform decision-making is continually evolving, it is assumed that new research should be incorporated into reviews. In addition, other aspects relevant to the review might change, such as ideas about the cause of the illness, ways of dealing with any adverse effects of the intervention, changes to the methodology used to combine intervention effects, or other aspects of health care relevant to people making decisions about the intervention. To prevent the evidence, and other information in the review, from becoming out-of-date or misleading, recommendations are sometimes made about the frequency with which the evidence base needs to be updated. It is the policy of The Cochrane Collaboration that reviews should be assessed, and if necessary updated, every two years, or should have a commentary added to explain why this is done less frequently [2].

However, the decision about when to update a Cochrane review must account for various factors. Failure to update reviews soon enough may cause decision makers to act on out-of-date information. On the other hand, reviews that are updated too soon may represent a waste of effort and resources [3] or introduce bias [4]. For example, systematic reviews with very few studies are particularly susceptible to 'time lag bias', which occurs when trials with positive results are published more quickly than those with negative or null results. A further danger of updating too frequently is that repeated significance tests can lead to inflated Type I Error. Constant updating could see positive results of meta-analyses purely by chance [5].

Several investigations of Cochrane reviews have examined the updating process. Chapman and colleagues [3] suggested that over three years, only a small percentage of updated reviews actually resulted in a change in conclusion. Koch [6] extracted and analysed the date fields of all reviews from *The Cochrane Library *Issue 1, 2002, to determine whether or not Cochrane reviews are being updated. He found that 68% of reviews (867 of the 1268) examined did not provide the date when new studies were found or the date of any amendments to the review authors' conclusions, and was unable to determine what proportion of reviews had actually been updated as opposed to simply edited. Higgins [7] examined all reviews in *The Cochrane Library *Issue 4, 1998. He found that 65 out of 481 reviews had gained at least one additional study since first appearing. Examination of the summary statistic of the primary outcome measure of these updated reviews revealed that statistical significance changed over time in just five reviews. Bastian and Doust [8] examined all reviews tagged as 'updated' in *The Cochrane Library *in 2003. Although this should be an indication that the review has changed substantively enough to warrant rereading [2], they found that it was often difficult to identify what had changed in the review or even which reviews had new data and/or changed conclusions.

The objective of this descriptive study is to describe the changes that occurred in a cohort of Cochrane systematic reviews over a four year period.

## Methods

### Development of a cohort of updated systematic reviews

All completed systematic reviews in the Cochrane Database of Systematic Reviews (CDSR) Issue 2, 1998 formed the original cohort of reviews for this project. The latest version of each of these reviews was then identified in CDSR Issue 2, 2002. The Cochrane Collaboration policy is that each review should be updated every two years, thus we chose a four year period to allow for as many reviews as possible to be updated.

Cochrane reviews give the date when the search for studies for inclusion was carried out. A review was defined as updated if a new search had been carried out between the two issues of CDSR examined, or the number of studies included in the meta-analysis of the primary outcome had changed.

Descriptive information was extracted from these pairs of reviews to determine the changes that had occurred during the four years. The information extracted included a determination of whether the review had been updated, whether it included new studies, or whether the original review had been withdrawn, replaced, merged with another review or split into multiple reviews. We also extracted the intervention summary statistic and its confidence interval for the primary outcome from each review. One member of the project team completed the data extraction. For validation purposes, a second member completed a quality assurance procedure on a 10% random sample of the data.

For the rest of the project, we examined only reviews that had been updated with the inclusion of additional studies.

### Determination of a changed conclusion

Cochrane reviews contain a section called 'Reviewers' Conclusions' where the authors discuss the implications of the review for practice and the implications for research. Both these sections of the conclusions were examined for change. The updated version of each review was compared to the original and any changes to conclusions were categorised by two investigators independently. Changes were classified as follows: no change; minor change (changes in style or wording that do not alter the substance or meaning of a section); and major change (changes that alter the substance or meaning of a section or alter the interpretation) [9]. Reviews that were judged as having a major change were categorised as having a changed conclusion. We did not independently assess or verify the review authors' conclusions, but instead relied on their interpretation of the results.

### Selection of the primary outcome measure

A primary outcome measure was identified in each of the reviews in Issue 2, 1998 using a pre-specified rule adapted from Higgins [7]. The primary outcome was determined from the review as either that stated by the authors as the primary outcome of interest or the first one listed under the 'Objectives' section of the review. If none was mentioned then we used the first outcome listed under the 'Types of outcome measures' subheading. If these approaches failed we used mortality.

### Statistical methods

#### Ratio of confidence intervals

For each review we calculated the width of the CI for the primary outcome in 1998 and 2002. When the summary statistic was an odds ratio or relative risk, the width of the CI was calculated on the natural log scale.

Relative precision of the original and updated review was calculated as the ratio of the width of the CI in 2002 to the width of the CI in 1998. To estimate the mean ratio and its precision, ratios were natural log transformed. This distribution was very skewed, and it was decided that bootstrapping would be a more appropriate method to calculate CIs rather than relying on large sample assumptions. Five thousand bootstrapped data sets were created using simple random sampling with replacement. For each of these data sets the mean of the logged ratios was calculated. The 2.5^th ^and 97.5^th ^percentiles of the distribution of estimated means were used to produce a 95% CI. These estimates were then back transformed to the original scale.

Logistic regression was used to examine if an increase in the precision of the CI in 2002, compared to 1998, was associated with a change in conclusion of the systematic review. An increase in precision being defined as a narrowing of the width of the CI in 2002 compared to 1998.

#### Determination of significance change

We examined whether a change in statistical significance of the summary statistic of the primary outcome was associated with a change in conclusion of the systematic review, using an exact 95% CI [10] for a difference in proportions.

All statistical analyses were performed using Stata 8.1 (StataCorp 2003. Stata Statistical Software: Release 8.1. College Station, TX: Stata Corporation.).

## Results

A total of 377 complete reviews were published in CDSR Issue 2, 1998. Thirty one Cochrane Collaborative Review Groups contributed to these reviews, with the greatest representation (33%) from the Cochrane Pregnancy and Childbirth Group.

**Figure 1:**
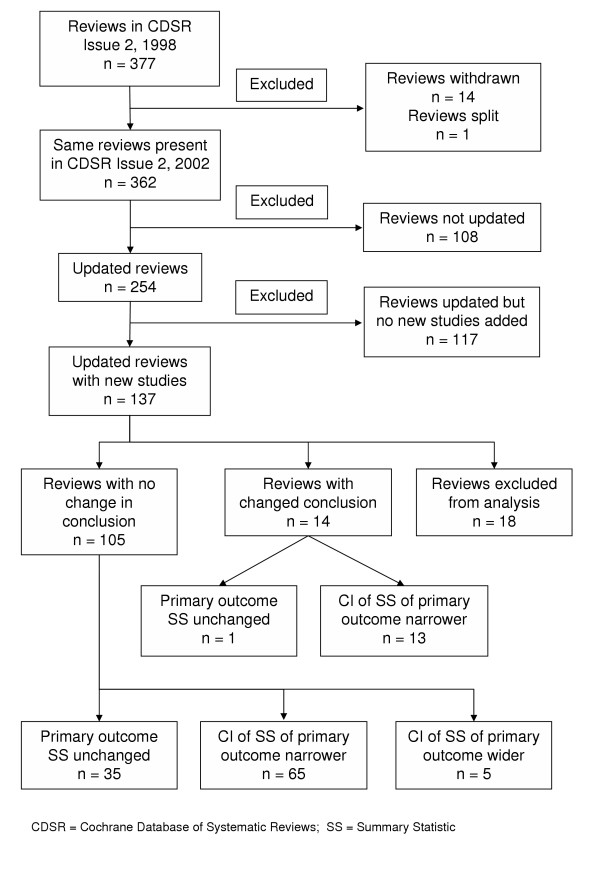
The status of all the reviews from CDSR Issue 2, 1998 in CDSR Issue 2, 2002.

Figure 1 outlines the status in CDSR Issue 2, 2002 of all the original reviews from CDSR Issue 2, 1998. During this time period, 14 reviews were withdrawn and one was split, leaving 362 reviews that were present in both issues of CDSR. Of these 362 reviews, approximately one third (38%) had been updated with new included studies, one third (32%) had re-run searches but included no new studies, and 30% had not been updated at all. The median number of studies per review increased from 5 (range 0 to 72) in 1998 to 6 (range 0 to 108) in 2002. Of the 254 updated reviews with and without new studies included, only 23 (9%) had a changed conclusion.

Of the 137 reviews updated with new studies, 18 reviews were excluded from our analysis because a summary statistic for the primary outcome was not available in one or both versions of the review, for example, if the review authors decided that the results of the included studies were not suitable for meta-analysis. Nine of these reviews had a change of conclusion and nine were unchanged. For the reviews with an unchanged conclusion, seven of the nine did not have a summary analysis available in either versions of the review, and two changed their research question resulting in a change in primary outcome. In the nine reviews with a changed conclusion, three did not have a summary analysis available in either version of the review and in four reviews either the research question changed or the outcomes examined changed resulting in a change in conclusion. In the remaining two reviews with a changed conclusion, a meta-analysis was not possible in the 1998 version and was then subsequently possible in the 2002 version.

This left 119 reviews in which new studies had been added to the updated review and for which data were available from the meta-analysis of the primary outcome from both 1998 and 2002. Further analysis was conducted only on these reviews to determine the effects of updating.

### Ratio of confidence intervals

**Figure 2:**
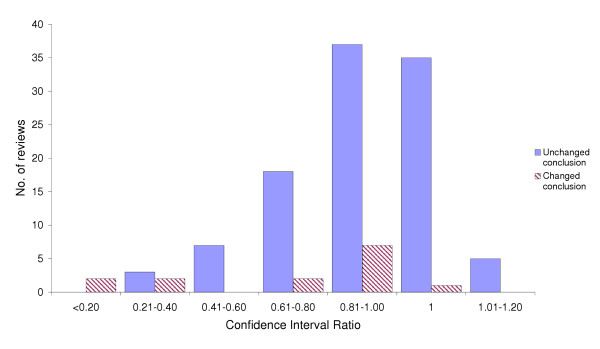
Ratios of confidence intervals of the summary statistic of the primary outcome of Cochrane reviews from Issue 2, 2002 to Issue 2, 1998.

Relative precision of the review pairs has been determined as a ratio of the width of the CI in 2002 to the width of the CI in 1998. For 85 of the 119 reviews (71%), the width of the CI around the primary outcome changed by less than 20% as a result of adding new studies (Figure 2). Of these, the width of the CI increased, remained the same, or decreased in five, 36, and 44 of the updated reviews respectively. For the five reviews with widened CIs, two had the same number of studies but had re-extracted the data from their original studies and in the other three reviews the updated meta-analysis led to only a small change in the precision (ratio range 1.04–1.11). The mean ratio of the width of the CI in 2002 to the width of the CI in 1998 was 0.81 (95% CI; 0.75, 0.86). For reviews with unchanged conclusions (n = 105) and changed conclusions (n = 14), this ratio was 0.85 (0.81, 0.89) and 0.56 (0.36, 0.81), respectively.

An increase in the precision of the CI in 2002 compared to 1998 (that is, a decrease in the width of the CI in 2002), increased the odds of a change in conclusion of the systematic review. In particular, for each percentage increase in the precision of the CI in 2002, the odds of a change in conclusion were 3.3% (95% CI; 1.0%, 5.6%) higher than the previous odds. Therefore, for a 19.1% increase in precision, as was observed on average between 1998 and 2002, the odds of a change in conclusion were 85.7% (95% CI; 21.1%, 184.6%) higher than those of no change in precision.

### Determination of significance change

Of the 119 reviews, 11 summary statistics of the primary outcome changed statistical significance between 1998 and 2002. Five changed from significant (p < 0.05) to non-significant (p ≥ 0.05), while the remaining six changed from non-significant to significant.

For the 11 reviews where there was a change in statistical significance of the summary statistic for the primary outcome, four reviews changed conclusion (36.4%). Of the 108 reviews where there was no change in the statistical significance, 10 reviews changed conclusion (9.3%). The difference in these percentages was 27.1% (95% CI; 0.7%, 60.3%).

Ten of the 14 reviews that changed conclusion did not have a change in significance of their summary statistic for the primary outcome. For eight of these reviews, the change in conclusion was related to a change for an outcome other than the primary outcome. Of the remaining two reviews, the change in conclusion in one was related to a sub-group analysis, and for the other review, the 1998 analysis was significant but based on only one small trial, and a further trial confirmed this in the 2002 review leading the review authors to be firmer in their conclusions.

## Discussion

The updating of Cochrane reviews has become the focus of some research in recent years [3,5-8,11]. Our study has examined in detail the impact of the updating process on the conclusions of a cohort of Cochrane systematic reviews over four years.

After this four year period, most (70%) reviews had been updated and of these, over half (54%) included new studies. A third of reviews (30%) had not been updated, with no indication that any new search for studies had been carried out. From the updated reviews, we estimated that 9% had changed conclusion. Concerns around a lack of updating for some reviews may not be justified considering the small percentage of updated reviews that resulted in a changed conclusion.

It is possible that the percentage of reviews that changed conclusion may differ in the reviews that had not been updated. The percentage would be overestimated, if for example, review authors were more likely to update their review given they had knowledge of new studies with conclusions that may alter the review conclusion. Conversely, the percentage would be underestimated, if under the above scenario, review authors were less likely to update their review because, for example, of workload issues. However, we believe this estimate is reasonable since the percentage of reviews with changed conclusions in those not updated would have to be quite different to that of the reviews that were updated to substantially change the overall estimate.

We compared reviews where the conclusion remained the same to those where the conclusion had changed. Factors predictive of a change in review conclusions were a decrease in the width of the CI in the updated review and a change in significance of the summary statistic of the primary outcome. These factors are of limited value in predicting which reviews should be updated, since they are only known after the update has taken place. A large percentage (50%) of the eighteen reviews excluded from our analysis had a change in conclusion. In the majority of these reviews, the change in the conclusion was due to a change of the research question.

The cohort of reviews selected for inclusion in this study was from the 1998 CDSR. It is possible that examination of a more recent cohort of reviews may provide different results. This may occur if, for example, the percentage of updated reviews was different, or the sizes and types of randomised controlled trials included have changed over time.

The process of updating systematic reviews is not unique to Cochrane reviews. The CDSR does, however, provide a unique opportunity to investigate this process due to the Cochrane Collaboration's policy to update reviews every two years. We are not aware of another study that has addressed the issue of updating in non-Cochrane reviews. The results we have found may also apply to non-Cochrane reviews, but we cannot be sure of this without further investigation. There may be reasons that non-Cochrane reviews are more likely to be updated and their conclusions change than Cochrane reviews. For example, non-Cochrane reviews may be carried out in clinical areas that are rapidly changing, and researchers may not want to go through the full Cochrane review process in order to get their review published more quickly. Systematic reviews in these clinical areas may be more likely to change conclusion.

Future research in this area should aim to establish the suitability of the current policy of The Cochrane Collaboration of updating every review every two years. This should provide a platform for a more empirically based set of recommendations about the updating of evidence in systematic reviews. In the meantime, there may be valid reasons to update a review more or less often than the current two year policy. Consequently, an approach that assesses the priority for updating individual reviews may be more appropriate in determining when reviews should be updated than a blanket time-bound policy. Other reasons for updating, such as the inclusion of a new intervention or outcome, and/or new methods for systematic reviews, will also need to be considered.

## Conclusion

The updating of Cochrane reviews consumes considerable time and resources and in many cases may not change the conclusion or lead to a more precise conclusion. Our study does not provide evidence to support or refute the current policy of The Cochrane Collaboration of updating reviews every two years. However, concerns around less frequent updating may not be justified in light of our results. A priority-setting approach for all reviews may be more appropriate than a rigid time-based approach.

## Competing interests

The Australasian Cochrane Centre is funded by the Australian Commonwealth Department of Health and Ageing and supported by Monash University. All authors are employed by the Australasian Cochrane Centre. SG is a member of the Cochrane Collaboration Steering Group.

The views expressed in this paper represent those of the authors and are not necessarily the views or the official policy of The Cochrane Collaboration (unless otherwise stated and referenced).

## Authors' contributions

SDF participated in the design of the study and extracted data. SM participated in the design of the study and extracted data. JEM performed the statistical analysis. SEG oversaw the study and participated in its design and coordination. All authors contributed to drafting of the manuscript, approved the final manuscript and are guarantors.

## Pre-publication history

The pre-publication history for this paper can be accessed here:


